# 20 years of crystal hits: progress and promise in ultrahigh-throughput crystallization screening

**DOI:** 10.1107/S2059798323001274

**Published:** 2023-02-27

**Authors:** Miranda L. Lynch, M. Elizabeth Snell, Stephen A. Potter, Edward H. Snell, Sarah E. J. Bowman

**Affiliations:** a Hauptman–Woodward Medical Research Institute, 700 Ellicott Street, Buffalo, NY 14203, USA; b Department of Materials Design and Innovation, The State University of New York at Buffalo, Buffalo, NY 14203, USA; c Department of Biochemistry, Jacobs School of Medicine and Biomedical Sciences at The State University of New York at Buffalo, Buffalo, NY 14023, USA; University of Western Australia, Crawley, Australia

**Keywords:** macromolecular crystallization, macromolecular crystallography, crystallization facilities, high-throughput crystallization, crystallization screening

## Abstract

Diffraction-based structural methods contribute a large fraction of the biomolecular structural models available, providing a critical understanding of macromolecular architecture. These methods require crystallization of the target molecule, which remains a primary bottleneck in crystal-based structure determination. The National High-Throughput Crystallization Center at Hauptman–Woodward Medical Research Institute has focused on overcoming obstacles to crystallization through a combination of robotics-enabled high-throughput screening and advanced imaging to increase the success of finding crystallization conditions. This paper will describe the lessons learned from over 20 years of operation of our high-throughput crystallization services. The current experimental pipelines, instrumentation, imaging capabilities and software for image viewing and crystal scoring are detailed. New developments in the field and opportunities for further improvements in biomolecular crystallization are reflected on.

## Abstract 2

The crystallization facility at Hauptman–Woodward Medical Research Institute, now the National High-Throughput Crystallization Center, has served the structural biology community for over two decades. The lessons learned from our high-throughput crystallization services, current operations and capabilities are described, together with a vision for the future of crystallization.

## 1. Introduction

Structural biology has long been a cornerstone of biological investigation, providing critical frameworks for understanding basic biological processes such as energy transduction and metabolism, as well as probing disease states and advancing drug discovery. The COVID-19 pandemic has underscored the central role played by macromolecular structures in the fight against disease (Lynch *et al.*, 2021 ▸). There are several tools available for determining or predicting the structures of biomolecules, ranging from spectroscopy (NMR) and microscopy (cryoEM) to computational methods (*RoseTTAFold* and *AlphaFold*2) and diffraction-based methods (microED, X-ray crystallography, X-ray free-electron lasers *etc.*). Even in the shadow of the cryoEM resolution revolution and recent advances in machine-learning-driven computational approaches to structure, macromolecular X-ray crystallography (MX) remains the dominant technique in the determination of three-dimensional structures, accounting for close to 90% of structures deposited in the Protein Data Bank (PDB; Burley *et al.*, 2020 ▸). The download statistics for PDB data in 2020 and 2021 underscore the central role that structures provide as a driving force in science (Berman *et al.*, 2007 ▸; https://www.wwpdb.org/stats/download). In 2020, close to 682 million files were downloaded from the PDB website, which corresponds to 1 873 193 files downloaded on average *per day*. These numbers have only grown: in 2021 there were over 719 million downloads during the year, which corresponds to close to two million files downloaded per day.

The role of MX in structural studies has recently been observed in real time as the COVID-19 pandemic has unfolded, with crystal-based structures informing on key steps in the development of vaccines and therapeutics, as well as providing details regarding viral biology. Despite this pre-eminent role of crystallography, the field continues to be hampered by one critical requirement: the formation of a crystal. Despite decades of effort, finding conditions in which macromolecular crystals form remains a key bottleneck in these structural efforts (Fazio *et al.*, 2014 ▸; Rosa *et al.*, 2020 ▸; Lynch *et al.*, 2020 ▸). The Crystallization Center at the Hauptman–Woodward Medical Research Institute (HWI) has been and remains at the forefront of providing automation, imaging and expertise to assist in the search for crystallization conditions, and is remarkably successful in this role.

The High-Throughput Crystallization Screening Center at HWI began operation in early 2000 using commercial liquid-handling robotics and developing in-house imaging platforms for a unique high-throughput (HT) experimental setup (Luft *et al.*, 2001 ▸). The initial formation and the first decade of operations have been described, including the selection and subsequent updates of the specific chemical cocktails used in each well of the HT screen (Luft *et al.*, 2001 ▸, 2003 ▸; Koszelak-Rosenblum *et al.*, 2009 ▸; Luft, Snell *et al.*, 2011 ▸). In July 2021, the Center received NIH NIGMS National Resource funding, establishing the National High-Throughput Crystallization Center (HTX Center) as a National Resource. This funding will enable the HTX Center to continue as a state-of-the-art resource for users, providing access to a centralized facility for crystallization expertise, training and instrumentation that are not typically available at independent laboratories. Here, we describe the considerable developments and improvements made over the past decade, the current operations, the lessons learned over the last 20 years, how to take advantage of the HTX Center and our vision for the future.

## 2. History and development of high-throughput crystallization screening at HWI

The HTX Center at HWI has been in continuous operation for over 20 years and has provided high-throughput crystallization screening services to over 2000 user laboratories for over 18 000 macromolecular samples to date. The HTX Center was an active component of the National Institutes of Health (NIH) Protein Structure Initiative (PSI), a NIH NIGMS-funded multiphase, multi-institutional structural genomics project that was active between 2000 and 2015 (Norvell & Berg, 2005 ▸, 2007 ▸). The PSI served as a concerted effort for widespread structural knowledge determination. The goal of the endeavor was to generate a large volume of protein structural information, often of challenging targets, for thousands of proteins and to contribute methods and protocols to enhance protein production and structure determination (Montelione, 2012 ▸; Michalska & Joachimiak, 2021 ▸). The first two phases of the program generated more than 5000 structures that were deposited in the PDB, with the final phase PSI:Biology contributing nearly 2000 additional structures (totaling 6920 over the duration of the project; Berman *et al.*, 2009 ▸; https://cdn.rcsb.org/sbkb/). While the total number of structures generated represents a relatively small fraction of the PDB, the PSI has had long-term impact through the discovery of novel folds and structural domains, as well as new Pfam families and subfamilies. The PSI also laid critical groundwork for significant methods development in robotics and automation, on which many modern crystallization laboratories depend. The HTX Center played a major role as one of the specialized centers designated under the PSI proposal and provided crystallization screening for several projects supported by the PSI, including the Northeast Structural Genomics (NESG) Consortium, the Structural Genomics of Pathogenic Protozoa (SGPP) Consortium, the Membrane Protein Structural Biology Consortium and the Center for High-Throughput Structural Biology Consortium. This exposed the HTX Center to a broad range of biological samples, helping to develop and improve crystallization screening processes over that period (Luft, Newman *et al.*, 2014 ▸). Success rates for producing crystal hits in the HTX Center were quantified during the PSI initiative and range from 27% (SGPP) to 47% (NESG) for PSI-associated samples (Snell, Luft *et al.*, 2008 ▸). For comparison, an internal study of 96 distinct macromolecules conducted at the time demonstrated a success rate of 51% (Snell, Lauricella *et al.*, 2008 ▸). A more recent analysis of NESG results revealed a positive crystal identification rate of 52%, with an overall rate of 21% for successful crystallographic models produced from crystals identified in the HTX Center screen (Snell, 2021 ▸). These success rates are comparatively high and reflect the accumulated experience in screening crystals at the HTX Center over two decades of operation.

The more than 18 000 different biomolecular samples have generated crystallization screening data on over 27 million screening experiments, and we have collected over 200 million images showing the progression of crystallization over time on these samples. In the HT pipeline at the HTX Center we have developed sample-handling protocols that ensure the integrity of samples that researchers from around the world mail to us (typically via overnight delivery). Each sample is set up in our unique HT 1536-well microassay plate with automated liquid-handling robots, enabling the precise and reproducible screening of a wide range of conditions using minimal sample volume and minimal crystallization reagents. The HTX Center provides two chemically diverse and carefully designed 1536-reagent crystallization assays: one for soluble proteins (Luft *et al.*, 2003 ▸) and one for membrane proteins (Koszelak-Rosenblum *et al.*, 2009 ▸). Both experimental screens were developed at HWI and use the microbatch-under-oil (MBO) crystallization method (Chayen *et al.*, 1992 ▸) with a high Saybolt viscosity paraffin oil. The historical development of the 1536 conditions used in the soluble screen has been described (Luft *et al.*, 2003 ▸). The membrane screen was developed based on experimentally determined phase boundaries for 11 different detergents (Koszelak-Rosenblum *et al.*, 2009 ▸). The HT membrane screen has only recently been modified in a small number of wells due to changes in the commercial screens. All changes between screen generations for both the soluble screen and the membrane screen are reflected in updated composite lists of chemical cocktails for each condition in each well and are available for current and previous generations.

After a sample has been set up in the 1536-well microassay plate, a robust imaging schedule is employed with state-of-the-art imaging instruments to monitor each of the 1536 wells for crystal growth over a six-week time window. Lysozyme samples are run monthly at both 23 and 14°C as controls, providing a rigorous and robust way to assess oil and cocktail delivery, cocktail precipitation issues and the performance of both the liquid-handling robotics and the imagers. These monthly quality-control experiments have enabled us to establish best practices for how long cocktails can be stored for subsequent use. Our goal is <1% error overall for all steps of the pipeline, and the controls ensure the rapid identification of any potential problems in the HT operations.

In its historical implementation in the HTX Center, the MBO technique was used to minimize the negative impact of dehydration during the experiment both in the sample-dispensing stage (which takes ∼10–15 min for the entire 1536-well plate) and during the six-week experimental time. MBO has the additional advantage of providing precise information on the equilibrium crystallization conditions that occur upon sample–cocktail mixing, which makes scale-up and optimization more straightforward. We find the optimization of crystallization conditions initially determined using the MBO method to be highly reproducible. The MBO technique has been evaluated, in terms of outcome success, in a study of 679 protein samples submitted to the HTX Center (Price *et al.*, 2009 ▸). In this study, 23.1% (157 of 679 samples) yielded structures that were deposited in the PDB after optimization from the initially identified crystal hits, while 5.7% (39 of 679 samples) only yielded low-diffraction quality crystals (Price *et al.*, 2009 ▸). In other words, 80% of the 196 samples for which crystallization conditions were identified in the HT 1536-condition screen were successfully optimized into high-diffraction quality crystals. Further, shifting from MBO to vapor diffusion (in either sitting-drop or hanging-drop format) is often effective (Chayen, 1998 ▸; Baldock *et al.*, 1996 ▸), although we find in some cases that HT MBO to optimized MBO has a higher success rate in scale-up and optimization experiments. Guidelines for converting conditions from microbatch to vapor diffusion and vice versa are available (Chayen, 1998 ▸). Experiments using plates from the HTX Center have also shown that the HT 1536-well microassay plates can be used for *in situ* X-ray diffraction data collection (Bruno *et al.*, 2016 ▸). It is also possible to harvest crystals directly from the HT 1536-well microassay plates (Luft, Grant *et al.*, 2014 ▸).

## 3. High-throughput screening: current experimental operations

An effective method to determine initial chemical conditions for protein crystallization is to chemically sample conditions that have proven to be effective for the crystallization of other proteins (Rosa *et al.*, 2020 ▸; Lynch *et al.*, 2020 ▸). This approach was used in the HTX Center to design the two chemically complementary sets of 1536 chemical cocktails (one for soluble proteins and one for membrane proteins), the experimental outcomes from which can be used to identify initial crystallization conditions and to guide crystal optimization (Luft, Wolfley *et al.*, 2011 ▸). Broadly, each 1536-condition HT screen contains 2–3 subsets of cocktails: (i) cocktails prepared in-house, (ii) commercially available screens and (iii) commercially available screens or reagent stock solutions that have been significantly modified in-house to improve their performance in batch crystallization experiments. All chemical conditions in the HT 1536-condition screen are composed of the cocktail mixed with the protein sample solution in a 1:1 ratio.

**Figure 1:**
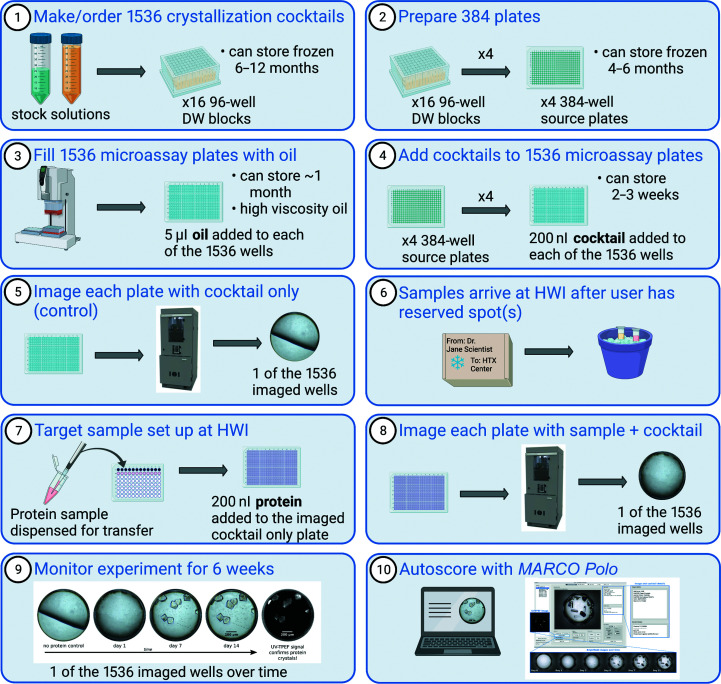
Schematic showing an overview of the steps in high-throughput crystallization screening experiments at the HTX Center: initial preparation of the setup (steps 1–5), initiation of the experiment upon receiving sample (steps 6–8) and experiment monitoring and assessment (steps 9–10). The bottom of each 1536 well is 0.9 mm in diameter (shown in steps 5, 8, 9 and 10).

For HT crystallization screening, our pipeline ensures robust operations and reproducibility, and efficient identification of potential crystallization conditions (Fig. 1 ▸). For the HT screens, the cocktails are reformulated 1–2 times per year and stored for 6–12 months in 96-well deep-well (DW) blocks at −20°C to maintain chemical integrity. Cocktails are generated from commercial reagents and supplies as much as feasible, but many of the conditions that we use are generated in-house. Specifically, 46.9% (720 of 1536 conditions) of the cocktails in the soluble screen are prepared in-house by the dilution of concentrated stock solutions of salts, buffers and polymers using a Formulatrix Formulator 16. In the membrane screen, 87.5% (1344 of 1536 conditions) of the cocktails are generated in-house. Commercial screens are purchased and used as supplied for 31.3% (480 of 1536 conditions) of the cocktails in the soluble screen and for 12.5% (192 of 1536 conditions) of the cocktails in the membrane screen. Finally, in the soluble screen, 21.8% (336 of 1536 conditions) of the cocktails are modified in-house from commercially prepared Hampton Research screens. Lists of the cocktails used for each generation of the 1536-condition soluble and membrane screens are available to download from the HTX Center website and are also linked as metadata to all software available for viewing crystallization experiments.

The 96-well DW blocks are formatted into 384-well source plates every 3–6 months using an Integra ViaFlo 384 equipped with a 96-channel pipetting head. Four 96-well DW blocks are used to stamp out each 384-well source plate such that quadrants are filled sequentially (A1 from 96-well DW1 is delivered to A1 in 384-well plate 1, A1 from 96-well DW2 is delivered to B1 in 384-well plate 1 *etc.*). The 384-well source plates are stored at −20°C for 4–6 months. HT 1536-well microassay plates are prepared 1–2 times per month in advance of receiving samples from users, as several steps are required to prepare the experimental plate prior to addition of a user sample. The HT 1536-well Imp@ct microassay plates used in the HTX Center were developed in collaboration with Greiner Bio-One specifically for use in our HT crystallization screening. Each of the 1536 wells has an opening with a diameter of 1.7 mm that tapers to a circular flat well bottom with a diameter of 0.9 mm. The optimized well geometry ensures that crystallization drops are located in the center of the well bottoms. The plates are made with a transparent polyolefin material designed to have low birefringence and limited UV background; the thin bottom is critical for automated imaging.

The number of 1536-well microassay plates prepared with oil and crystallization cocktails each month is based upon the number of reservations tallied from reservation requests, plus an additional percentage to allow flexibility for the acceptance of late samples. White mineral/paraffin oil (100%, Saybolt viscosity 340–365 at 100°F; EMD PX0045-3) is first delivered to the 1536-well microassay plates using an Integra ViaFlo 384 equipped with a 384-channel pipetting head. The 1536-well microassay plates are inverted to enable imaging through the most efficient optical path; specialized adapters are used to image these inverted plates. High-viscosity oil in the HT MBO setup is essential to ensure that the liquid in each of the wells of the 1536-well plate is not displaced via gravity while imaging occurs. Additionally, the specific high-viscosity oil used in the HTX Center shows the lowest dehydration when measured over time.

Cocktails are delivered to the oil-filled 1536-well microassay plates from the set of four 384-well source plates using an Art Robbins Griffin or Platemate (both are equipped with 384-syringe heads). The four 384-well plates are stamped out into the 1536-well plate such that quadrants are filled (A1 in 384-well plate 1 to A1 in the 1536-well plate, A2 in 384-well plate 1 to A3 in the 1536-well plate *etc.*). The delivery protocols from both the 96-well DW blocks to the 384-well plates and from the 384-well plates to the 1536-well crystallization screening plate ensure that all 1536 cocktails are delivered to the correct well. Each well receives 200 nl of cocktail solution, and the delivery protocol is similar for both the soluble and membrane screens. The syringe-based liquid-handling systems use scripts with reduced rates of aspiration and dispensing, added lag times and trial dispenses to accurately and precisely deliver the wide range of physical chemical properties that are represented in the cocktail solutions. The plates are then centrifuged to ensure that the drops are resting at the bottom of the well under the layer of oil. All plates are imaged prior to the addition of protein sample; each plate is inspected prior to sample delivery to ensure that the cocktail has been delivered.

After a user has reserved a spot in the screening queue, they ship the protein sample to the HTX Center. Sample-shipping requirements vary depending on the nature of the sample. As users have more specific knowledge of their samples, we encourage them to send their samples on dry ice, in dry shippers, on wet ice or on cold packs, choosing the condition in which the sample will be most stable and most likely to reach the HTX Center intact. For each 1536-well microassay plate crystal screening experiment, 500 µl of sample is required. We recognize the variability of sample-handling needs and encourage interactions between users and the HTX Center to ensure sample integrity upon arrival and during any subsequent sample-handling steps. Samples are typically centrifuged prior to setup. Observations are recorded regarding the protein solution before and after centrifugation to note any precipitation of the sample, as well as to record the clarity and color of the solution. The protein is manually delivered to a row of 12 wells of a 96-well source plate. Currently, two of the Platemate liquid-handling robots have custom-made 12-syringe heads to deliver 200 nl protein to each well in the 1536-well microassay plate. The HTX Center is also investing in a SPT Labtech Mosquito for protein delivery to the HT microassay plate. The Mosquito will replace the end-of-life Platemate robots in early 2023. The HTX Center is receptive to trialing new instrumentation that may fit into the operational pipeline and enhance the success of crystallization outcomes.

Dispensing the user sample to a 1536-well microassay plate takes ∼10–15 min, making the actual time that the protein is handled very minimal. Following the addition of protein, the 1536-well microassay plate is centrifuged to merge the protein and cocktail solutions under the oil and is then imaged (day one image). The rapid setup of crystallization screening experiments reduces the time for protein degradation; this is a significant advantage of the HTX Center protocols. Initial testing of the Mosquito in our pipelines is promising for the use of both a lower volume of sample and less time for sample setup. We are encouraged by the promising preliminary indications in this improved pipeline and are formalizing a study to quantify the improvements.

## 4. Imaging and computing infrastructure and pipelines

Imaging of crystallization screening experiments is a crucial aspect of HT pipelines, as it provides an efficient means of identifying crystals in large-scale screens and contributes valuable data on the crystallization process (Liu *et al.*, 2008 ▸). The HTX Center has state-of-the-art imaging instrumentation that is uniquely designed to detect macromolecular crystals. The workhorse of the imaging pipeline is the Formulatrix Rock Imager 1000 with SONICC (RI-1000), which enables enhanced crystal imaging using second-harmonic generation (SHG) and ultraviolet two-photon excited fluorescence (UV-TPEF) microscopies (Madden *et al.*, 2011 ▸; Haupert & Simpson, 2011 ▸). We also have a Formulatrix Rock Imager 54 (RI-54) in a 14°C cold room devoted to crystallization studies. The Rock Imagers are used to obtain brightfield (BF) images for each well of the 1536-well microassay plates over time, using adaptors specifically designed for the inversion of the plates for imaging. The BF capabilities of the RI-1000 and RI-54 are greatly enhanced relative to the previously utilized in-house-built imaging tables. We began using the RI-1000 for SONICC imaging in 2015 and for all brightfield imaging in 2020; the RI-54 was installed in 2020. Optics have improved substantially in the past 20 years, and the Rock Imagers both enable continuous zoom optics so that multiple images at different *z*-heights are obtained. BF images are taken prior to sample addition, at day one and at weeks 1, 2, 3, 4 and 6. Imaging all 1536 wells in the HT screening plates takes 40–45 min using BF.

In addition to BF imaging, the HTX Center also uses multi-photon imaging techniques for enhanced crystal detection. At one time point in the course of a HT screening experiment, SHG and UV-TPEF images are taken of each well in the 1536-well microassay plate using the RI-1000. SHG microscopy is a nonlinear optical process in which two photons from a high-intensity femtosecond laser are converted to a frequency-doubled response (second-harmonic frequency) in certain materials. In ordered systems such as crystals, this results in coherent addition and strong SHG signal generation; SHG imaging is therefore highly selective for crystalline material (Haupert & Simpson, 2011 ▸; Kissick *et al.*, 2011 ▸). UV-TPEF is comparable to standard ultraviolet fluorescence (Madden *et al.*, 2011 ▸; Padayatti *et al.*, 2012 ▸). Aromatic side chains of amino-acid residues absorb in the UV wavelength region, with tryptophan being the primary fluorophore. The two-photon excited fluorescence signal is detected in the reflected direction via a photomultiplier tube detector in the RI-1000. In tandem with BF imaging, these advanced imaging methods provide information, specifically on whether the materials are likely to be protein-containing (via UV-TPEF) and crystalline (via SHG). Imaging all 1536 wells in the HT screening plates takes approximately 4.5 h using both UV-TPEF and SHG modalities. The HTX Center is unique in making these imaging capabilities available to external users.

**Figure 2:**
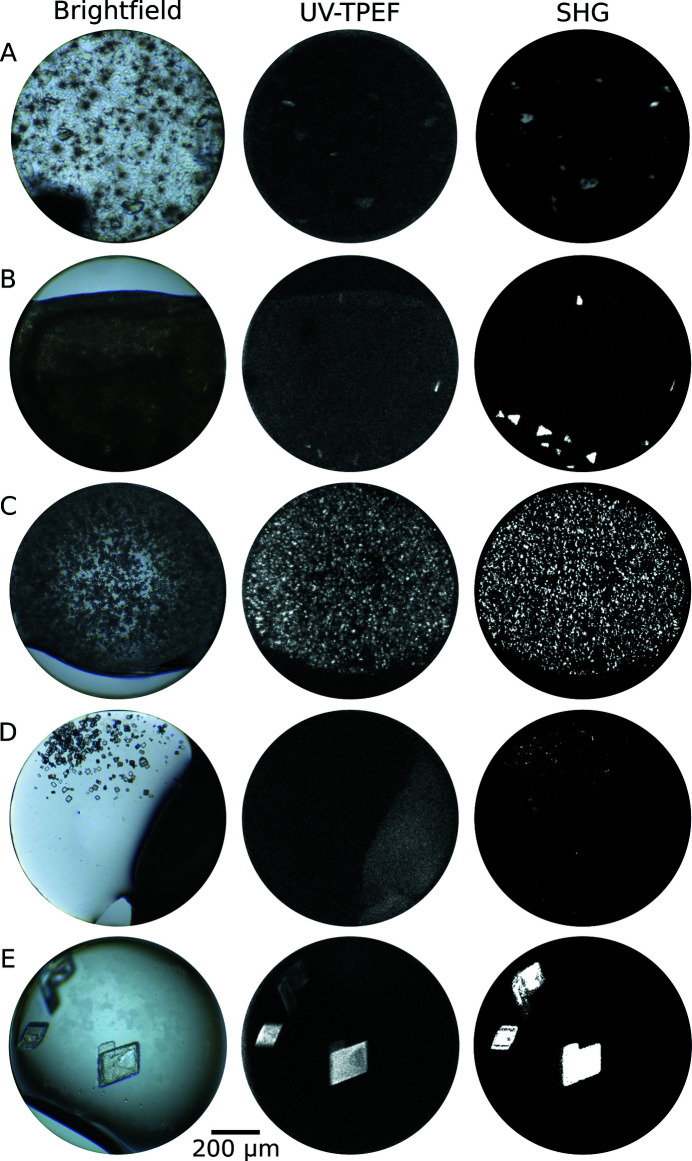
Images of crystallization experiments from the HTX Center using the RI-1000 with SONICC. Each row is from a single well (of 1536) imaged at one time point with BF (left), UV-TPEF (middle) and SHG (right).

The improvements enabled by the RI-1000 with SONICC are profound. Using BF images alone for protein crystal detection can lead to a number of pathologies, many of which are exemplified in the first column of images in Fig. 2 ▸. Crystals can be too small to see or hidden under precipitate, resulting in false negatives. Failure to detect a crystal that is present is problematic since it is so difficult to generate protein crystals to begin with. Crystals that are detected with BF can be composed of salt from the crystallization buffer and not contain protein at all, resulting in false-positive signals. These are especially challenging outcomes because of the subsequent time, effort and materials expended to optimize protein crystals. Combining BF images with UV-TPEF and SHG images provides solutions to all of these difficulties. Fig. 2 ▸ shows examples from HT crystal screening images from the HTX Center using the three image modalities. In row A the BF image has precipitate obscuring the visible protein crystals. The large bright spots in the UV-TPEF and SHG images pinpoint the presence and location of the protein-containing crystals. Rows B and C show heavy precipitate, which is revealed to consist of small crystals in the UV-TPEF and SHG images. In row D the BF image shows clear crystals, but the corresponding lack of UV-TPEF signal and the presence of SHG signal indicate that these crystals are most likely to be salt and are not protein-containing at all. Row E has positive identification of protein crystals in all three images. The sequence of images in Fig. 2 ▸ demonstrates the utility realized by combining the unique capabilities of each imaging modality into a single overall assessment of image information. It is clear that UV-TPEF and SHG imaging capabilities enhance the interpretation and quality of information when trying to identify positive crystallization outcomes.

When the 1536-well microassay plates are not actively being imaged in the RI-1000 or the RI-54, the plates are kept either in the Rock Imager hotel [temperature regulated at 23°C (RI-1000) or 14°C (RI-54)] or in temperature-controlled incubators or rooms at 4, 14 or 23°C. Temperature is a critical variable that dramatically impacts protein solubility and crystallization outcomes. Upon request, the 1536-well microassay experiment plate can be shipped overnight without disrupting the crystals or other experimental outcomes, which has positive implications for the possibilities of *in situ* crystallization options (Bruno *et al.*, 2016 ▸). When requested, plates are shipped to researchers in an insulated Styrofoam container with gel packs to stabilize the temperature.

The high-throughput nature of sample processing at the HTX Center necessitates a robust computing and quality-control infrastructure geared to maintain two critical functionalities: operations management and data management. Operations management comprises all aspects of sample receipt and tracking, scheduling equipment maintenance and tracking of quality and output metrics for operations. The HTX Center has maintained uninterrupted operation and has screened many thousands of samples since it became operational. This has been accomplished through strict adherence to standard operating procedures, preventative maintenance protocols and meticulous monitoring of the robotic liquid-handling instruments. We use a protein control (hen egg-white lysozyme; Hampton Research) to test all operations of the HTX Center on a monthly basis, allowing quality control for crystallization reagents, robotic liquid-handling instruments and the imaging equipment. These procedures have enabled a very extended useful equipment lifetime for all of the instruments in use in the HTX Center.

The HTX Center employs a LIMS system connected to the sample-submission interface. In addition, the experiment pipelines for crystallization screening and optimization generate a large volume of image data, with concomitant metadata on the experiments. We have pipelines in place for image storage, and employ FTP to provide the experimental data to users. We also provide two different image-viewing software interfaces for users to view their crystallization images. The legacy interface, *MacroscopeJ*, is an in-house-developed Java-based software specifically constructed for the unique 1536-well screening platform used by the HTX Center and is still available to users. We have also recently developed and released a new graphical user interface (GUI), *MARCO Polo*. This open-source software is a Python-based comprehensive menu-driven user interface for viewing and scoring crystallization images (Holleman *et al.*, 2021 ▸). *MARCO Polo* displays crystallization images linked to experimental metadata and implements the *MAchine Recognition of Crystallization Outcomes* (*MARCO*) scoring algorithm. The *MARCO* algorithm was developed by a consortium of academic and industrial partners including Google Brain (Bruno *et al.*, 2018 ▸). A training set of nearly half a million scored BF crystallization images from a number of crystallization facilities worldwide (including the HTX Center) was used to train a deep convolutional neural network to classify images into four classes. The *MARCO* algorithm has been reported to achieve an accuracy of 94.5% (Bruno *et al.*, 2018 ▸), a level of success exceeding human scorer accuracy, which is estimated at 84% (Fusco *et al.*, 2014 ▸). The *MARCO Polo* software is under active development at the HTX Center and currently enables users to view different imaging types including SHG and UV-TPEF; further developments are in progress to also enable viewing of different plate formats using *MARCO Polo*.

## 5. Looking forward

The field of crystallography is dynamic and continues to grow as new experimental and measurement technologies are developed. Advances occurring at synchrotron facilities include enhanced detectors, higher brilliance and microfocused beams, improved remote-access options, room-temperature capabilities and novel sample-delivery methods. New diffraction techniques, such as serial synchrotron methods, X-ray free-electron lasers and microcrystal electron diffraction, are also enlarging the field and contributing vibrant new approaches to structural science. In the HTX Center, we have focused on finding initial crystallization conditions for over 20 years and have been at the forefront of developing these methods to be efficient, robust and scalable. We have worked with crystallization metadata from the HTX Center historical database, as well as data mined from the PDB, to try to detect patterns and to advance our understanding of the chemical crystallization space that yields protein crystals (Lynch *et al.*, 2020 ▸; Fusco *et al.*, 2014 ▸; Bruno *et al.*, 2014 ▸; Altan *et al.*, 2016 ▸). These internal metadata coupled with knowledge of the outcome have also been used to identify ligands that have previously been missed (Bruno *et al.*, 2014 ▸) and to adapt processes to the capabilities of new sources such as X-ray free-electron lasers (Luft *et al.*, 2015 ▸). The HTX Center also develops computational approaches that can be used by others, as shown by the *MARCO* algorithm for classifying crystallization outcomes (Bruno *et al.*, 2018 ▸) and its implementation into the *MARCO Polo* GUI (Holleman *et al.*, 2021 ▸).

A growing field entails the increasing needs of scientists using crystallography to address important biological questions. The HTX Center is committed to maintaining its position at the forefront of crystallization science by actively monitoring the shifting technological landscape, leveraging our wealth of experimental data to continue to improve our processes and pipelines, and expanding the range of our crystallization services. We do this through our critical contact with the user community, through our investment in cutting-edge instrumentation for imaging and crystal handling, and through our constant testing and development of method­ologies and protocols. In addition to maintaining state-of-the-art crystallization services for use by the community, we are also actively engaged in research on crystallization methods. New computational projects that are currently under way in the HTX Center include improved computational and machine-learning tools for better classification of crystallization outcomes, mining the enormous data resources on HTX Center operations to gain new insights into the physical process of biomolecular crystallization, and new image-analysis tools for crystal characterization and nanocrystal detection. The technological advances that enable the use of much smaller crystals for structural studies requires con­comitant development of new ways to detect and handle these small crystals. Current technological research projects include developing new approaches for tuning crystal growth, integrating novel and gentler sample-handling and transfer methods into crystallization pipelines, creating methods for *in situ* crystallization and quantifying information contributions from different imaging modalities. As a centralized and well equipped facility, we make the latest high-throughput methods, robotics and instrumentation easily available to a large number of scientists. Critically for us, there is reciprocity and synergy with our user community, whose scientific questions help to drive the development of new techniques and provide the impetus for new methodologies to be developed.
